# Poor sleep and decreased cortical thickness in veterans with mild traumatic brain injury and post-traumatic stress disorder

**DOI:** 10.1186/s40779-024-00557-0

**Published:** 2024-08-05

**Authors:** Murray J. Andrews, David H. Salat, William P. Milberg, Regina E. McGlinchey, Catherine B. Fortier

**Affiliations:** 1 Boston University Chobanian and Avedisian School of Medicine, Boston, MA 02118 USA; 2 Translational Research Center for TBI and Stress Disorders (TRACTS), VA Boston Healthcare System, Boston, MA 02130 USA; 3 Department of Psychiatry, Harvard Medical School, Boston, MA 02138 USA; 4 Department of Psychiatry, Boston University School of Medicine, Boston, MA 02130 USA; 5 Neuroimaging Research for Veterans Center, VA Boston Healthcare System, Boston, MA 02130 USA; 6 Anthinoula A. Martinos Center for Biomedical Imaging, Boston, MA 02129 USA; 7 Geriatric Research, Educational and Clinical Center (GRECC), VA Boston Healthcare System, Boston, MA 02130 USA

**Keywords:** Trauma, Brain injury, Sleep, Veterans, Cortical thickness

## Abstract

**Background:**

Poor sleep quality has been associated with changes in brain volume among veterans, particularly those who have experienced mild traumatic brain injury (mTBI) and post-traumatic stress disorder (PTSD). This study sought to investigate (1) whether poor sleep quality is associated with decreased cortical thickness in Iraq and Afghanistan war veterans, and (2) whether these associations differ topographically depending on the presence or absence of mTBI and PTSD.

**Methods:**

A sample of 440 post-9/11 era U.S. veterans enrolled in the Translational Research Center for Traumatic Brain Injury and Stress Disorders study at VA Boston, MA from 2010 to 2022 was included in the study. We examined the relationship between sleep quality, as measured by the Pittsburgh Sleep Quality Index (PSQI), and cortical thickness in veterans with mTBI (*n* = 57), PTSD (*n* = 110), comorbid mTBI and PTSD (*n* = 129), and neither PTSD nor mTBI (*n* = 144). To determine the topographical relationship between subjective sleep quality and cortical thickness in each diagnostic group, we employed a General Linear Model (GLM) at each vertex on the cortical mantle. The extent of topographical overlap between the resulting statistical maps was assessed using Dice coefficients.

**Results:**

There were no significant associations between PSQI and cortical thickness in the group without PTSD or mTBI (*n* = 144) or in the PTSD-only group (*n* = 110). In the mTBI-only group (*n* = 57), lower sleep quality was significantly associated with reduced thickness bilaterally in frontal, cingulate, and precuneus regions, as well as in the right parietal and temporal regions (β = −0.0137, *P* < 0.0005). In the comorbid mTBI and PTSD group (*n* = 129), significant associations were observed bilaterally in frontal, precentral, and precuneus regions, in the left cingulate and the right parietal regions (β = −0.0094, *P* < 0.0005). Interaction analysis revealed that there was a stronger relationship between poor sleep quality and decreased cortical thickness in individuals with mTBI (*n* = 186) compared to those without mTBI (*n* = 254) specifically in the frontal and cingulate regions (β = −0.0077, *P* < 0.0005).

**Conclusions:**

This study demonstrates a significant relationship between poor sleep quality and lower cortical thickness primarily within frontal regions among individuals with both isolated mTBI or comorbid diagnoses of mTBI and PTSD. Thus, if directionality is established in longitudinal and interventional studies, it may be crucial to consider addressing sleep in the treatment of veterans who have sustained mTBI.

## Background

Many US veterans who were deployed to Iraq and Afghanistan during Operation Enduring Freedom (OEF), Operation Iraqi Freedom (OIF), and Operation New Dawn (OND) returned from combat with serious physical and psychological trauma. Among this population, there is a high prevalence of sleep disruption [1]. The disturbance of sleep is commonly observed even among veterans without a diagnosis of post-traumatic stress disorder (PTSD) [2]. Recent research conducted on civilian populations has uncovered links between disrupted sleep, neural health, and neurodegenerative conditions. For example, inadequate sleep has been associated with neurodegenerative disease [3, 4] as well as reduced brain volumes in specific regions [5–13]. In a large sample taken from the UK Biobank, it was found that longer-than-recommended sleep duration was linked to an overall decrease in grey matter volume globally, while shorter-than-recommended sleep duration was associated with a reduction in cortical surface area globally, particularly affecting the frontal, cingulate, and occipital lobes [14]. Furthermore, a study involving cognitively intact older adults discovered an association between self-reported poor sleep quality and global measures indicating abnormalities in brain microstructure [15]. This apparent relationship between sleep patterns and brain structure has also been established in the context of obstructive sleep apnea, where daytime sleepiness has been correlated with lower cortical thickness in the frontal, temporal, and parietal lobes [16].

Additionally, mild traumatic brain injury (mTBI) with a prevalence of approximately 15%, and post-traumatic stress disorder (PTSD) with a prevalence ranging from 6.2 to 12.9%, are highly prevalent among veterans of the wars in Iraq and Afghanistan [17–19]. Previous studies have reported associations between cortical thickness and both mTBI and PTSD. Lower volume in limbic and paralimbic structures such as the hippocampus, cingulate, and insular cortex has been identified in civilian [20] and veteran [21, 22] populations with PTSD. Similarly, decreased cortical thickness in the frontal and temporal lobes has been observed in civilian [23] and veteran [24, 25] populations with mTBI.

Previous studies have examined the neural correlations between sleep and brain volume in either civilian [26–28] or veterans [29] with mTBI and/or PTSD diagnoses, revealing a consistent association between reduced cortical thickness and poor sleep quality within disease-specific regions mentioned above. To our knowledge, there is currently no existing study that has compared the disease-specific topographical relationships between subjective sleep quality and cortical thickness in individuals with PTSD and mTBI. Therefore, it remains unclear whether the relationship between sleep quality and cortical thickness occurs in the specific context of disease, or if poor sleep quality is associated with decreased cortical thickness regardless of diagnosis. Given the high comorbidity between TBI and PTSD in veteran populations [19, 30, 31], it is important to characterize the overlapping neurobiological mechanisms underlying each disorder. Furthermore, although the directionality of the relationship between sleep and cortical thickness remains uncertain, longitudinal studies in cognitively healthy individuals have established associations between poor sleep at baseline and increased longitudinal cortical thinning [5, 32]. By further elucidating these relationships in the context of specific diseases, valuable insights can be gained for clinical management. For example, if poor sleep is found to cause increased longitudinal cortical thinning in a disease-specific manner, then interventions targeting sleep may be used as a disease-modifying treatment. Conversely, if poor sleep quality occurs as a result of cortical thinning, assessing sleep quality could aid in estimating disease severity and interventions targeting sleep may be employed as symptomatic relief.

We investigated the structural neural correlations of sleep quality in a large sample of OEF/OIF/OND veterans with and without PTSD and mTBI in the Translational Research Center for Traumatic Brain Injury and Stress Disorders (TRACTS) Cohort at VA Boston. To do so, we examined the associations between sleep quality, as measured by a self-report questionnaire, and cortical thickness assessed through structural magnetic resonance imaging (MRI) in individuals diagnosed with mTBI, PTSD, comorbid mTBI/PTSD, or neither condition. Additionally, we compared the topography of the resulting maps. Based on previous research identifying reduced regional thickness associated with mTBI and PTSD, our hypothesis posited that sleep quality would be related to cortical thickness in a disease-specific manner, such that poor sleep would be more strongly correlated with decreased cortical thickness in frontal regions in individuals with mTBI, while poor sleep would be more strongly correlated with decreased cortical thickness in limbic and paralimbic regions in individuals with PTSD.

## Materials and methods

### Participants

Data from 440 veterans of OEF/OIF/OND enrolled in the Translational Research Center for Traumatic Brain Injury and Stress Disorders (TRACTS) study from 2010 to 2022 were examined. TRACTS is a Veterans Affairs (VA) Rehabilitation Research and Development (RR&D) supported TBI Center of Excellence (CoE) at VA Boston Healthcare System that examines physiological, cognitive, psychological, and neurological functioning in post-9/11 Veterans, with an emphasis on traumatic brain injury (TBI) and stress disorders. All participants provided written informed consent and study procedures were approved by the Institutional Review Board of Human Studies Research at the VA Boston Healthcare System. Detailed information about methodology and inclusion/exclusion criteria has been previously described [33]. Briefly, all participants had been deployed to a warzone at least once and were recruited during military events in the Boston area. Individuals were excluded from participation if they had a history of serious illnesses unrelated to the study conditions (mTBI, PTSD), including a history of seizure disorder, cerebrovascular accident, myocardial infarction, diabetes; or if they exhibited current active suicidal and/or homicidal ideation with intent, or plan requiring crisis intervention; or if they currently met Diagnostic and Statistical Manual of Mental Disorders **(**DSM)-IV diagnostic criteria for bipolar disorder, schizophrenia, or any other psychotic disorder (except psychosis not otherwise specified due to trauma-related hallucinations); or if they had cognitive disorder due to general medical condition other than TBI. Furthermore, given that this analysis was focused on discrete changes to the brain structure, individuals with any history of moderate or severe TBI were excluded from this specific analysis, as these cases typically involve more prominent brain damage which may have confounded our analyses. Finally, individuals were also excluded if they had any factors that potentially impacted neurological function or cognitive performance, or if they met significant psychiatric criteria.

### Clinical assessment

All participants underwent a psychiatric assessment conducted by a doctoral-level psychologist to determine if they met the criteria for PTSD and/or mTBI. The Clinician-Administered PTSD Scale (CAPS) for DSM-IV [34] was used to assess PTSD, while the Boston Assessment for TBI-Lifetime (BAT-L) [30] was utilized to evaluate mTBI. TRACTS is a longitudinal cohort study that began enrollment in 2010 under the purview of DSM-IV. The assessment battery was updated to DSM-5/CAPS-5 when available but given the longitudinal cross-sectional sample drawn for this analysis, only CAPS-IV was available for all participants. We found high diagnostic agreement (92.9–95.4%), sensitivity (94.4–98.2%), specificity (91.7–92.8%), positive (89.5–93.0%), and negative (95.7–98.1%) predictive value, and interrater reliability (κ = 0.86–0.91) for CAPS-IV versus CAPS-5 in our sample of TRACT veterans [35]. The BAT-L is an extensively validated and widely used semi-structured clinical interview designed for diagnosing TBI across the lifespan in post-9/11 military veterans with particular attention to blast-related injury [30]. The BAT-L employs standard field criteria such as duration of loss of consciousness, posttraumatic amnesia, and altered mental status to classify TBIs into mild, moderate, or severe categories. Of the 440 participants, it was found that 110 individuals met the criteria for current PTSD based on symptoms experienced within the previous month without any history of military-related mTBIs, while 57 had suffered at least one military-related mTBI but did not meet the criteria for current PTSD. A further 129 individuals met the criteria for both PTSD and mTBI. The remaining group consisted of 144 individuals who did not meet the criteria for either condition and thus served as a comparison group. Depression was assessed using the depression scale from the Depression Anxiety Stress Scale (DASS), a validated tool for the assessment of depression [36, 37]. Sleep quality was evaluated using the Pittsburgh Sleep Quality Index (PSQI), a validated index for self-report sleep quality [38]. The PSQI is an 18-item questionnaire that rates 7 component scores (sleep duration, sleep disturbance, sleep latency, daytime disturbance, habitual sleep efficiency, sleep quality, and use of sleep medication) on a scale of 0 to 3. These scores are then summed to yield a global score ranging from 0 to 21, with higher scores indicating poorer sleep. Each participant in our study completed the PSQI at the time of their visit in order to assess sleep over the last month.

**Fig. 1:**
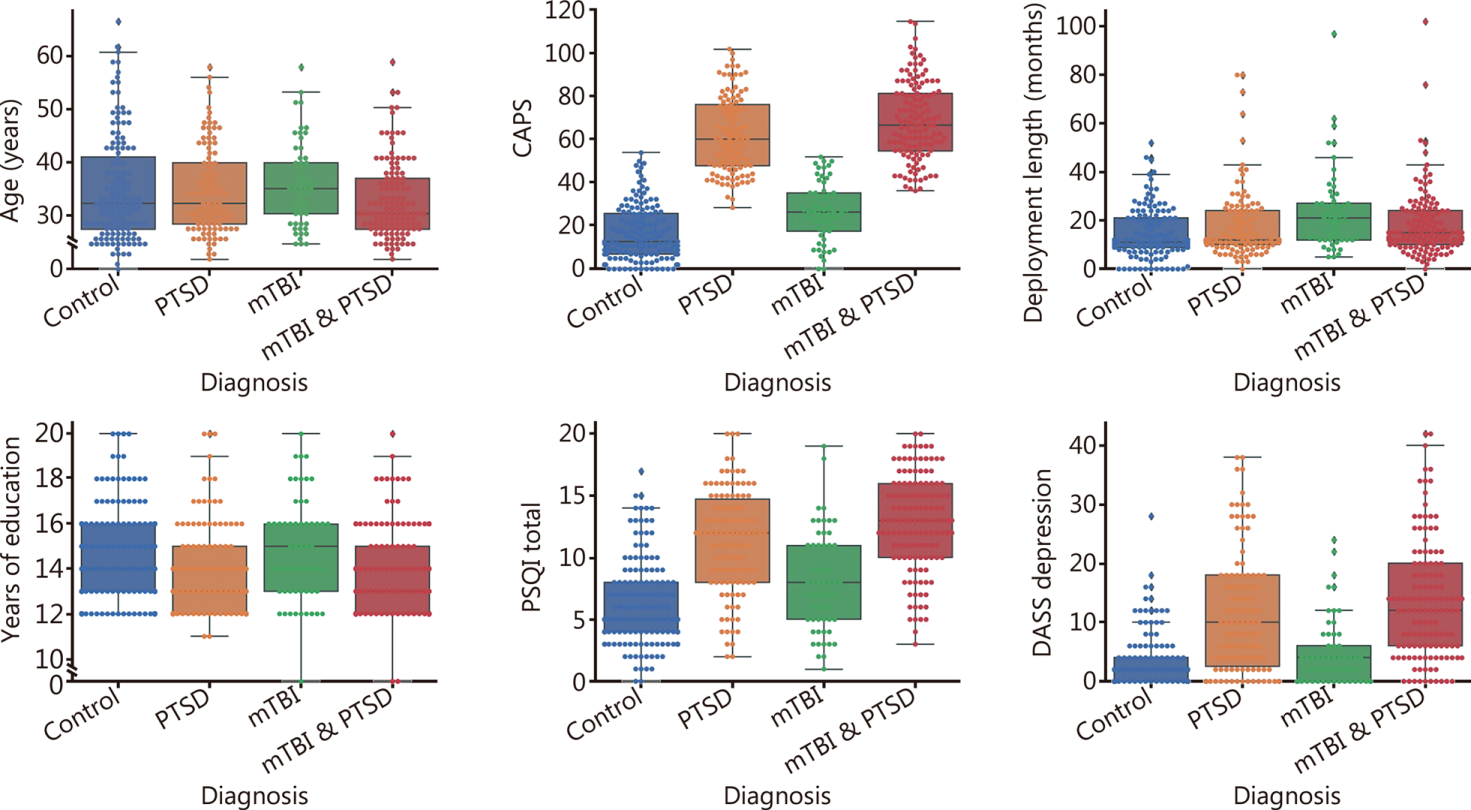
Box plots of participant demographics by diagnosis. Box plots of participant demographic information in individuals with post-traumatic stress disorder (PTSD), mild traumatic brain injury (mTBI), both (mTBI & PTSD) and neither (Control). CAPS Clinician-Administered PTSD Scale, PSQI Pittsburgh Sleep Quality Index, DASS Depression Anxiety Stress Scale

Demographic information on these groups is summarized in Table 1. Differences between groups were assessed using ANOVA. Box plots of these variables are shown in Fig. 1.

**Table 1:** Participant characteristics

Characteristic	Whole group (*n* = 440)	PTSD-only (*n* = 110)	TBI-only (*n* = 57)	Comorbid TBI and PTSD (*n* = 129)	Neither TBI nor PTSD (*n* = 144)	*P-*value
Age (years, mean ± SD)^a^	33.2 ± 9.0	33.4 ± 8.5	34.8 ± 8.2	31.7 ± 7.5	33.8 ± 10.4	*P* = 0.1
Female [*n* (%)]^a^	43 (9.8)	15 (13.6)	1 (1.8)	8 (6.2)	19 (13.2)	*P* = 0.02
CAPS (mean ± SD)^a^	45.50 ± 28.91	63.46 ± 18.11	27.37 ± 13.97	70.26 ± 18.31	16.78 ± 13.22	*P* < 0.001
Deployment length in months (mean ± SD)^a^	17.72 ± 13.65	18.29 ± 14.74	23.67 ± 16.31	18.53 ± 13.70	14.21 ± 10.13	*P* < 0.001
Years of education (mean ± SD)^a^	14.20 ± 2.17	13.95 ± 2.08	14.84 ± 2.29	13.78 ± 2.03	14.53 ± 2.20	*P* = 0.002
PSQI (mean ± SD)^a^	9.69 ± 4.76	11.30 ± 4.16	8.00 ± 4.00	12.85 ± 3.89	6.31 ± 3.53	*P* < 0.001
DASS depression (mean ± SD)^a^	8.50 ± 9.36	11.62 ± 10.23	4.74 ± 5.69	13.54 ± 9.97	3.10 ± 4.58	*P* < 0.001
Race [*n* (%)]						*P* = 0.95
White	333 (75.7)	78 (70.9)	42 (73.7)	105 (81.4)	108 (75.0)	
Black	40 (9.1)	13 (11.8)	5 (8.8)	9 (6.0)	13 (9.0)	
American Indian	2 (0.5)	1 (0.9)	0 (0.0)	0 (0.0)	1(0.7)	
Asian	8 (1.8)	2 (1.8)	1 (1.8)	2 (1.6)	3 (2.1)	
Pacific Islander	2 (0.5)	0 (0.0)	0 (0.0)	1 (0.8)	1 (0.7)	
Other/Unknown	55 (12.5)	16 (14.6)	9 (15.8)	12 (9.3)	18 (12.5)	

*CAPS* Clinician-Administered PTSD Scale, *PSQI* Pittsburgh Sleep Quality Index, *DASS* Depression Anxiety Stress Scale, *PTSD* post-traumatic stress disorder, *mTBI* mild traumatic brain injury

^a^Used as a covariate in General Linear Model (GLM) analyses

### MRI acquisition and processing

Imaging was performed at the Neuroimaging Research for Veterans (NeRVe) Center, located at the Boston VA Healthcare Center in Jamaica Plain, MA. Of the 440 participants, 312 individuals were scanned using a Siemens 3 T Magnetom Trio scanner with a 12-radiofrequency channel head coil, while 128 participants were scanned using a Siemens 3 T Magnetom Prisma scanner with a 20-radiofrequency channel head coil. For each participant, two T1 structural magnetization-prepared rapid gradient-echo (MPRAGE) images were acquired and averaged to increase the signal-to-noise ratio. In the case that excessive motion was visually detected at the scanner console, an additional scan was obtained. Scans with excessive motion determined by visual inspection were flagged and excluded from analysis. The acquisition parameters remained consistent across both scanners (3D sequence, flip angle 7o, field of view 256 × 256 × 276 mm^3^, repetition time of 2530 ms, and slice thickness of 1 mm), except for echo time which differed slightly between scans acquired on the Trio scanner (3.32 ms) and those acquired on the Prisma scanner (3.35 ms). Notably, individuals scanned with Prisma exhibited significantly higher age compared to those scanned with Trio (37.12 years vs. 31.62 years, *P* < 0.0001), possessed more education (15.02 years vs. 13.87 years), had longer deployment durations (21.29 months vs. 16.26 months, *P* = 0.0004), but did not differ in any of the other variables examined. The inclusion of scanner type as a nuisance variable in analyses aimed to account for any possible variability introduced between scanners.

Images were processed using FreeSurfer version 7.2 to segment and parcellate each scan and reconstruct the cortical surface for subsequent cortical thickness analyses [39, 40]. Each reconstruction was inspected for quality and manually edited when needed. Cortical thickness was extracted as the closest distance from the gray/white boundary to the pial surface at each vertex [41]. Resulting thickness maps were then smoothed using a Gaussian kernel with a full width at half maximum (FWHM) of 20 mm.

### Statistical analysis

To determine the topographical relationship between subjective sleep quality and cortical thickness in each diagnostic group, we employed a General Linear Model (GLM) at each vertex on the cortical mantle. In this model, thickness was considered as the dependent variable while the global PSQI score served as the independent variable. Additionally, age, sex, years of education, CAPS total score, and DASS depression scale were included as covariates in each analysis. To account for multiple comparisons, a Monte Carlo simulation with 5000 iterations was utilized to correct all analyses at a vertex-wise and cluster-wise threshold of *P* < 0.05. In the analyses that revealed a significant association between global PSQI score and cortical thickness, further vertex-wise analyses were conducted using each PSQI subcomponent as the dependent variable. The extent of topographical overlap between the resulting statistical maps was assessed by calculating Dice coefficients for each pair of maps. The Dice coefficient provides a quantitative metric of spatial overlap ranging from 0 (no overlap) to 1 (complete overlap) [42], offering insight into potential shared neural architecture across different analyses. To examine the interactive effect of mTBI and PSQI on cortical thickness, a vertex-wise GLM was fitted with thickness as the dependent variable and the interaction between mTBI and PSQI as an independent variable with age, sex, years of education, CAPS total score, DASS depression scale, and comorbid PTSD diagnosis as covariates. Following vertex-wise analyses, the regions showing significant effects were used to create regions of interest (ROIs). The mean cortical thickness in these ROIs was then calculated for each participant. Finally, the mean cortical thickness within each ROI was utilized as a dependent variable in a GLM, with the global PSQI score serving as the independent variable. and age, sex, years of education, CAPS total score, and DASS depression scale as covariates.

## Results

### Associations between PSQI and cortical thickness

**Fig. 2:**
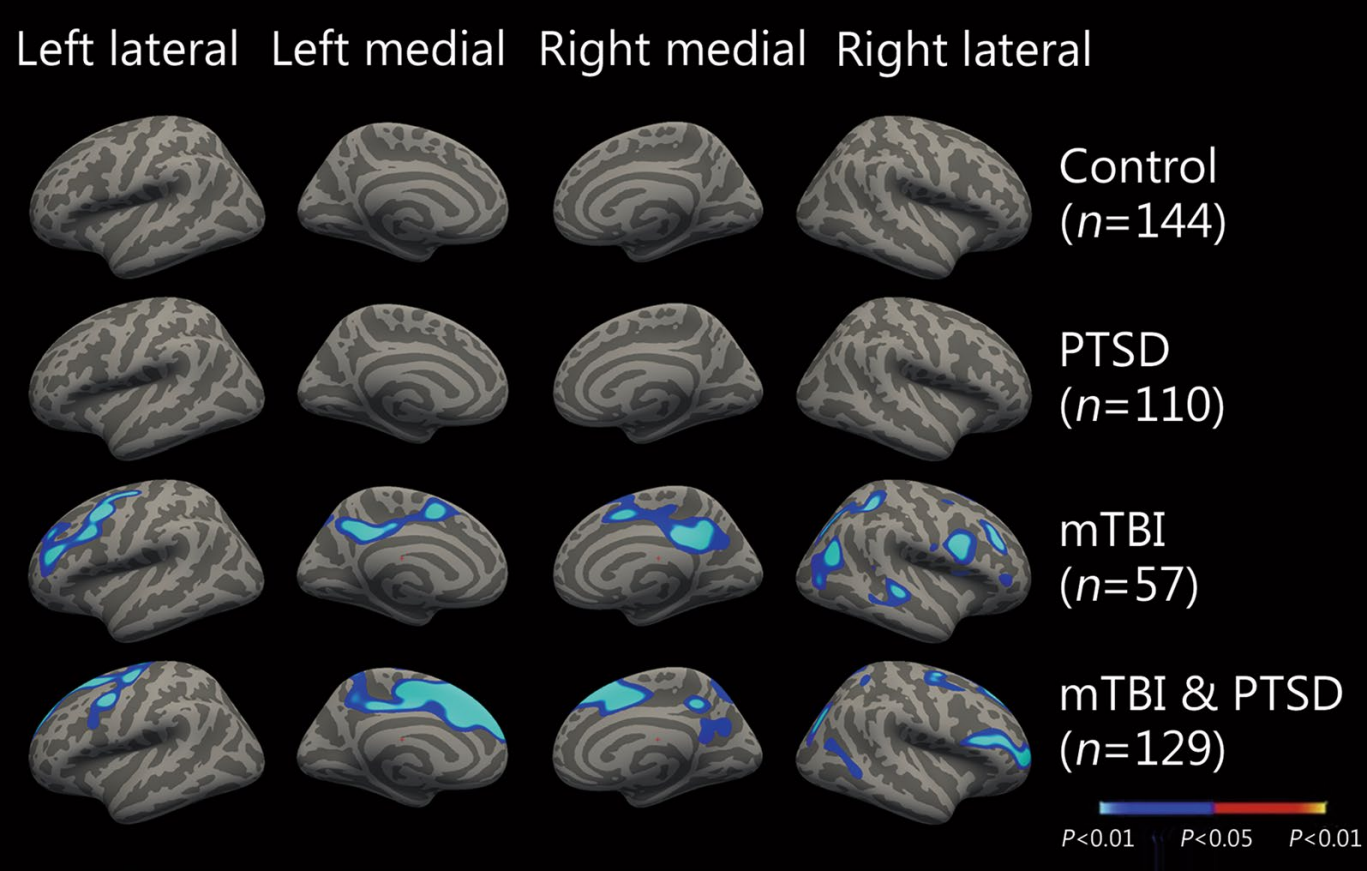
Magnetic resonance imaging (MRI) maps showing association of sleep with cortical thickness. Regional Associations between Pittsburgh Sleep Quality Index (PSQI) and cortical thickness in individuals with post-traumatic stress disorder (PTSD, *n* = 110), mild traumatic brain injury (mTBI, *n* = 57), both diagnoses (comorbid mTBI and PTSD, *n* = 129), and neither diagnosis (neither mTBI nor PTSD, *n* = 144). Blue indicates regions in which increased PSQI (poorer sleep) was significantly associated with decreased cortical thickness

Associations between PSQI and cortical thickness were examined in individuals with mTBI-only (*n* = 57), PTSD-only (*n* = 110), both diagnoses (*n* = 129), and neither diagnosis (*n* = 144) as depicted in Fig. 2. There were no significant associations between PSQI and cortical thickness in groups without mTBI or PTSD, or PTSD-only group. In mTBI-only group, a higher PSQI score indicating poor sleep quality was associated with reduced cortical thickness bilaterally in regions including superior frontal, rostral and caudal middle frontal, posterior/isthmus cingulate, precuneus, as well as right superior and inferior parietal and superior temporal regions. In comorbid mTBI and PTSD group, significant clusters were observed bilaterally in regions such as superior frontal, rostral and caudal middle frontal, precentral and precuneus, left posterior cingulate and caudal anterior cingulate, right superior and inferior parietal regions. There were no significant associations between cortical thickness and any of the PSQI subcomponents in mTBI-only group or comorbid mTBI and PTSD.

### Topography of the interactive effect of mTBI and PSQI on cortical thickness

**Fig. 3:**
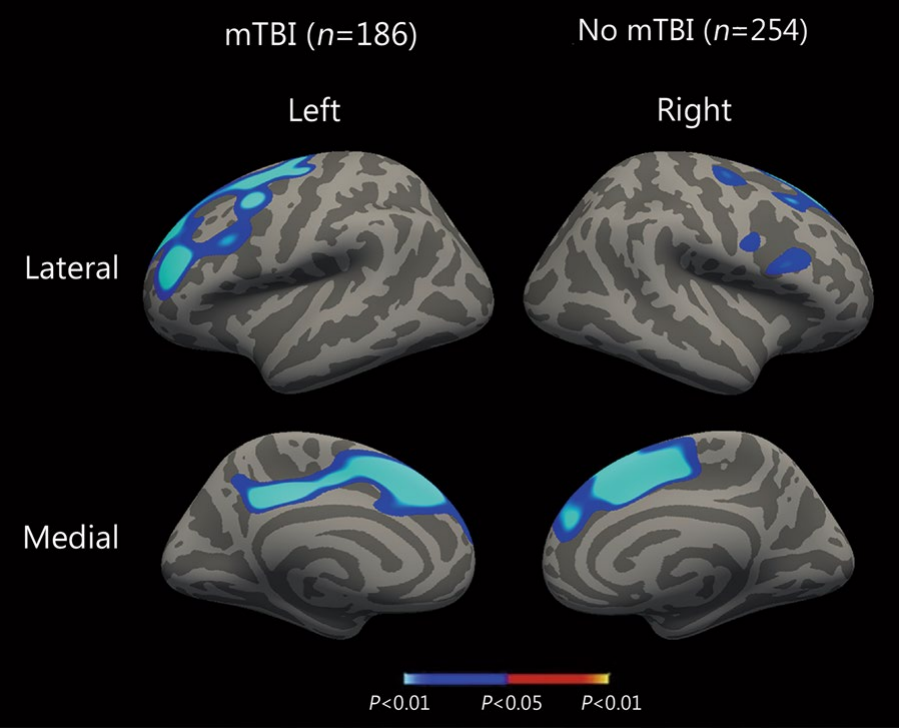
Magnetic resonance imaging (MRI) maps showing interactive effect of mild traumatic brain injury (mTBI) and Pittsburgh Sleep Quality Index (PSQI) on cortical thickness. Blue areas indicate significantly decreased cortical thickness with increased PSQI in individuals with mTBI (*n* = 186) compared to individuals without mTBI (*n* = 254), corrected for age, sex, Clinician-Administered post-traumatic stress disorder (PTSD) Scale and Depression Anxiety Stress Scale—Depression Subscale, and comorbid PTSD

Statistical maps illustrating the association between mTBI by PSQI interaction and cortical thickness are presented in Fig. 3. Blue areas indicate a significant interactive effect of mTBI diagnosis and PSQI on cortical thickness, indicating a more significant negative relationship between PSQI and cortical thickness in individuals with mTBI (*n* = 186) than in individuals without mTBI (*n* = 254). Significant effects were primarily detected in the frontal and cingulate regions.

### Comparison of the topography of regional associations (Dice scores)

Given the replication of the general effect between poor sleep quality and reduced cortical thickness in both mTBI-only and comorbid mTBI and PTSD groups, we investigated the degree of spatial overlap (replicability) of these effects. The Dice coefficients between mTBI-only and comorbid mTBI and PTSD groups were computed as 0.467 for the left hemisphere and 0.415 for the right hemisphere, indicating a moderate level of overlap.

### ROI-based analyses

**Fig. 4:**
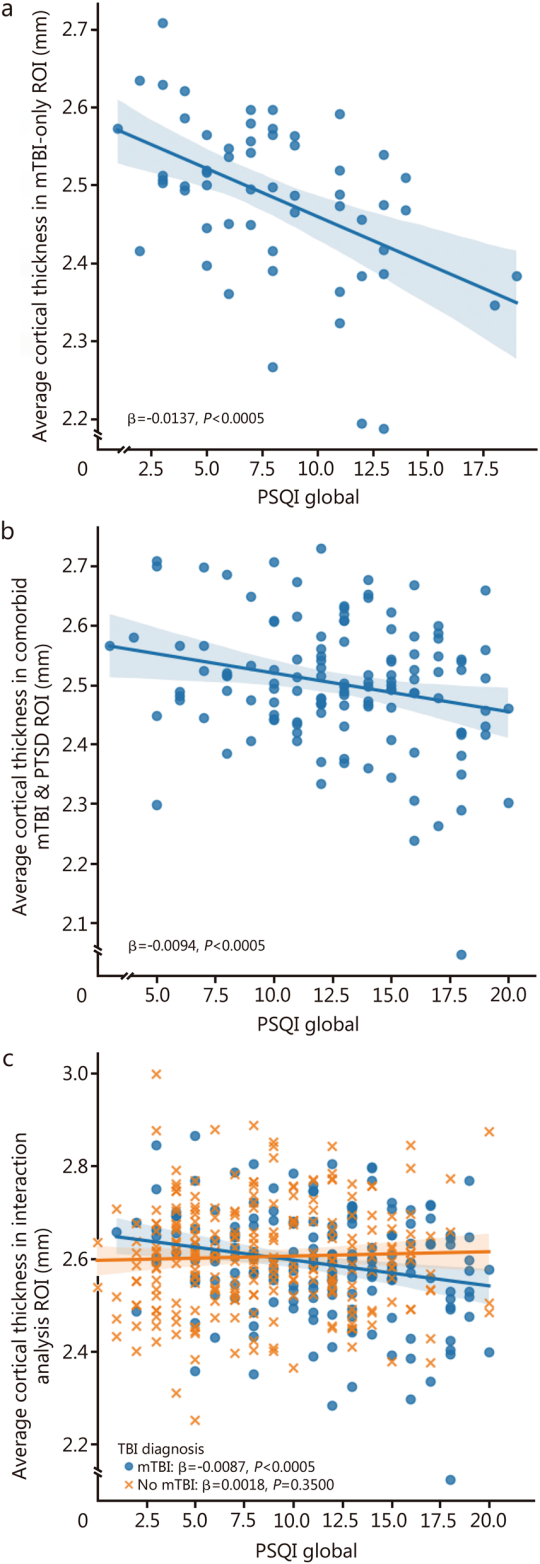
Associations between Pittsburgh Sleep Quality Index (PSQI) and cortical thickness in regions of interest (ROIs) generated from vertex-wise analyses. **a** Association between mean cortical thickness in the regions of interest (ROI) generated from the mild traumatic brain injury (mTBI)-only whole-brain analysis and Pittsburgh Sleep Quality Index (PSQI) in individuals with mTBI only (*n* = 57). **b** Association between mean cortical thickness in the ROI generated from the comorbid mTBI and post-traumatic stress disorder (PTSD) whole-brain analysis and PSQI in individuals with comorbid mTBI and PTSD (*n* = 129). **c** Association between mean cortical thickness in the ROI generated from the whole-brain interaction analysis and PSQI in individuals with mTBI (*n* = 186) and individuals without mTBI (*n* = 254)

The associations between PSQI and mean cortical thickness in the ROIs derived from the whole-brain analyses are shown in Fig. 4. In mTBI-only group (*n* = 57), PSQI was significantly associated with mean cortical thickness in the ROI generated from the exclusive mTBI-only whole-brain analysis (β = −0.0137, *P* < 0.0005). In the comorbid mTBI and PTSD group, a significant association was found between PSQI and mean cortical thickness in the ROI generated from the comorbid mTBI and PTSD whole-brain analysis (β = −0.0094, *P* < 0.0005). Furthermore, a significant relationship was identified between PSQI and cortical thickness in the ROI derived from the interaction analysis among individuals with mTBI (*n* = 186, β = −0.0087, *P* < 0.0005), while no such association was observed in individuals without mTBI (*n* = 254, β = 0.0018, *P* = 0.3500). Notably, there existed a significant interaction effect of mTBI diagnosis and PSQI on mean cortical thickness in the ROI generated from the interaction analysis (β = −0.0077, *P* < 0.0005).

## Discussion

The current study demonstrated a cross-sectional association between sleep quality measured by PSQI and cortical thickness. Specifically, it was found that poor sleep quality was associated with reduced cortical thickness in frontal and cingulate regions. Importantly, these regional relationships between PSQI and cortical thickness were observed in both individuals with comorbid mTBI and PTSD group, as well as those with mTBI only, suggesting that the relationship between mTBI, subjective sleep quality, and cortical thickness exists regardless of PTSD presence. The overlap in regions associated with poor sleep between mTBI-only and comorbid mTBI and PTSD groups highlights the importance of mTBI in this effect, as indicated by the Dice coefficients. No significant regional relationships were found in the PTSD-only group, further supporting this finding. Furthermore, interaction analyses revealed a significant interactive effect of mTBI and PSQI on cortical thickness, specifically indicating a more significant negative relationship between PSQI and cortical thickness in individuals with mTBI compared with those without mTBI.

The groups exhibited significant differences in some of the variables used as covariates. However, these differences were anticipated for post-9/11 era Veterans based on the presence or absence of their common comorbid conditions. Specifically, we expected a significant difference in CAPS score across the groups given that individuals with PTSD are classified by this score. Furthermore, the higher DASS depression scores observed in PTSD-only and comorbid mTBI and PTSD group were anticipated given the negative affect symptoms that are part of the constellation of PTSD symptoms. Finally, there is a notable disparity in PSQI scores between the groups, with higher scores seen in both PTSD-only and comorbid mTBI and PTSD groups compared with those with mTBI only. Considering sleep disruption is included in diagnostic criteria for PTSD, it is expected that more significant sleep disturbances would be present among individuals diagnosed with PTSD. It should also be noted that there were significant differences between scanner models regarding age, education level, and deployment length. However, since each analysis controlled for these variables individually as well as for scanner usage, these disparities should not significantly impact the results.

The regional relationships observed in our analyses were primarily limited to the frontal cortex and cingulate gyrus, with some involvement of the precuneus, as well as gyri in the parietal and temporal lobes. These specific brain regions align with previous studies investigating cortical thickness, which identified frontal gyri [24, 25] as regions associated with reduced cortical thickness in the context of mTBI. Furthermore, Lindemer et al. [22] found significant effects of mTBI on PTSD-related reduced cortical thickness in bilateral superior frontal regions in the TRACTS cohort. Our results provide support for prior research suggesting a potential role of the frontal cortex in sleep disturbance following mTBI. The underlying reason for the specific susceptibility of these regions remains unclear; however, it has been suggested that their anatomical location and interaction with skull structures make them more vulnerable to mechanical forces during TBI [43]. These results are also consistent with those reported by Chao et al. [29], who identified negative correlations between PSQI and thickness in various frontal regions, including the anterior cingulate cortex, among a group of Gulf War veterans independent of psychiatric diagnoses. However, they did not account for mTBI when conducting their analyses.

In this study, there was no significant association observed between PSQI and cortical thickness in the large sample of veterans with PTSD without comorbid mTBI. Previous research has reported associations between sleep quality and brain structure in non-military populations. Nardo et al. [28] identified a negative association between a score measuring sleep disturbances and grey matter volume in bilateral amygdalae, hippocampi, and right anterior cingulate cortex in a sample of 21 civilians with PTSD. Furthermore, Seo et al. [26] utilized polysomnography and actigraphy to examine relationships between sleep and cortical thickness in 77 trauma-exposed individuals with and without PTSD. They found that indicators of poor sleep were negatively correlated with cortical thickness in areas in the cingulate cortex, frontal lobe, and precuneus, while indicators of good sleep showed positive correlations in these regions. Multiple considerations should be made when evaluating the discrepancies between these findings and the results from our study. It is possible that physiological markers for assessing sleep are more sensitive than self-report measures (PSQI) for detecting such associations. The sleep disturbances score utilized by Nardo et al. [28] focused on insomnia and nightmares, while the PSQI provides a more comprehensive assessment of overall sleep quality. Therefore, these scales may offer different indices for evaluating sleep quality. Future studies investigating changes in PTSD-related sleep may consider alternative methods to assess sleep quality. Furthermore, the structural alterations related to disturbed sleep identified by Nardo et al. [28], overlap with previous research on PTSD-related structural changes, which have shown strong negative relationships between PTSD severity levels and thickness specifically within the amygdala and hippocampus [44, 45]. It is possible that sleep-related structural changes in these subcortical areas, which may be the most pronounced, might not have been detected by our study which primarily focused on cortical regions.

There were no significant associations between any of the PSQI subcomponents and cortical thickness in either the mTBI-only or the comorbid mTBI and PTSD groups. This may indicate that global sleep quality has a stronger correlation with cortical thickness than any specific sleep measure. However, it should be considered that the global PSQI score (0–21) captures a wider range of variation compared with the individual subcomponents (0–3), which may increase the likelihood of detecting correlations. Future studies should examine the relationship between cortical thickness and objective measures of sleep in these domains to determine potential specific implications.

The following limitations should be taken into consideration. As discussed above, our study utilizes the PSQI as a subjective measure of sleep quality. Furthermore, although the PSQI includes the use of sleep medication as a component, it fails to account for the utilization of other medications that may affect sleep and thus could have influenced the results of this study. It is plausible that objective measures would be more sensitive in detecting sleep changes among individuals with PTSD and mTBI. This study was also limited to cortical regions, thereby neglecting any associations between sleep and volume in deeper brain regions. Recent analyses conducted on the TRACTS cohort have demonstrated a correlation between poor subjective sleep quality and alterations in white matter, indicating the involvement of deeper brain structures in the relationship between sleep quality and brain structure integrity [46]. Furthermore, our study specifically examined military-related mTBI. Thus, the presence of mTBI outside of a military context may have impacted cortical size and affected our results. It is also worth noting that females were significantly underrepresented in this sample. Sex differences in the manifestations and neurobiology of TBI [47] as well as PTSD [48] have been previously established. Hence, the underrepresentation of females in this sample significantly limits the generalizability of these results. Minoritized groups were also inadequately represented in this sample population, which further restricts generalizability. Finally, while our cross-sectional study has identified an interactive association between mTBI, subjective sleep quality, and cortical thickness, the directionality underlying these relationships remains unknown.

Previous research on the neurobiology of sleep has suggested significant functional involvement of the medial frontal cortex and cingulate gyrus in sleep maintenance [49, 50], and Koenig et al. [51] identified a significant link between focal dorsomedial frontal damage and insomnia. Thus, it is possible that lower thickness in these regions associated with mTBI, as demonstrated here, may cause significant sleep disruption. Alternatively, rodent models have demonstrated the neuroprotective effect of sleep following axonal injury [52], while poor sleep after mTBI has been associated with worse neurocognitive outcomes in human studies [53–55]. Therefore, poorer post-mTBI sleep may exacerbate cortical thinning in regions affected by mTBI by interfering with normal recovery processes. Recent longitudinal analyses in the TRACTS cohort have shown that increased cortical atrophy is associated with more severe PTSD symptoms and a history of mTBI at baseline. It is possible that a combination of PTSD, mTBI, and poor sleep together contribute to increased longitudinal cortical thinning [56]. Future studies should focus on establishing causality in these relationships using longitudinal data.

## Conclusions

This study presents a specific association between poor subjective sleep quality and reduced frontal cortical thickness in veterans of OEF/OIF/OND who have experienced mTBI. This association is observed in individuals with only mTBI as well as those with mTBI and comorbid PTSD. These results underline the importance of considering sleep quality in the treatment of veterans who have suffered from mTBI, while also highlighting the necessity for future investigation into the relationship between sleep, brain structure, and function in the context of mTBI.

## Data Availability

The data is owned by the US Department of Veterans Affairs and therefore will not be made publicly available. Inquiries to access data can be made to the Translational Research Center for TBI and Stress Disorders (TRACTS) through the corresponding author.

## Abbreviations

BAT-L: Boston Assessment for TBI-Lifetime
CAPS: Clinician-Administered PTSD Scale
CoE: Center of Excellence
DASS: Depression Anxiety Stress Scale
FWHM: Full Width at Half Maximum
GLM: General Linear Model
MPRAGE: Magnetization-Prepared Rapid Gradient-Echo
MRI: Magnetic resonance imaging
mTBI: Mild traumatic brain injury
NeRVe: Neuroimaging Research for Veterans
OEF: Operation Enduring Freedom
OIF: Operation Iraqi Freedom
OND: Operation New Dawn
PSQI: Pittsburgh Sleep Quality Index
PTSD: Post-traumatic stress disorder
ROI: Regions of interest
RR&D: Rehabilitation Research and Development
TRACTS: Translational Research Center for Traumatic Brain Injury and Stress Disorders
VA: Veterans Affairs
